# Impairment of RAD17 Functions by miR-506-3p as a Novel Synthetic Lethal Approach Targeting DNA Repair Pathways in Ovarian Cancer

**DOI:** 10.3389/fonc.2022.923508

**Published:** 2022-07-18

**Authors:** Marina Bagnoli, Roberta Nicoletti, Monica Valitutti, Andrea Rizzo, Alessandra Napoli, Rafaela Montalvão De Azevedo, Antonella Tomassetti, Delia Mezzanzanica

**Affiliations:** Department of Research, Unit of Molecular Therapies, Fondazione IRCCS Istituto Nazionale dei Tumori, Milan, Italy

**Keywords:** miR-506-3p, ChrXq27.3 miRNA cluster, RAD17, cell cycle checkpoint inhibitors, DNA damage and repair, ovarian cancer

## Abstract

Epithelial ovarian cancer (EOC) remains the most lethal gynecological cancer and development of chemo-resistance is a major factor in disease relapse. Homologous recombination (HR) is a critical pathway for DNA double strand break repair and its deficiency is associated to a better response to DNA damage-inducing agents. Strategies to inhibit HR-mediated DNA repair is a clinical need to improve patients’ outcome. MicroRNA (miRNAs) affect most of cellular processes including response to cancer treatment. We previously showed that miR-506-3p targets *RAD51*, an essential HR component. In this study we demonstrated that: i) another HR component, *RAD17*, is also a direct target of miR-506-3p and that it is involved in mediating miR-506-3p phenotypic effects; ii) the impairment of miR-506-3p binding to *RAD17* 3’ UTR reverted the miR-506-3p induced platinum sensitization; iii) miR-506-3p/RAD17 axis reduces the ability of EOC cell to sense DNA damage, abrogates the G2/M cell cycle checkpoint thus delaying the G2/M cell cycle arrest likely allowing the entry into mitosis of heavily DNA-damaged cells with a consequent mitotic catastrophe; iv) RAD17 expression, regulated by miR-506-3p, is synthetically lethal with inhibitors of cell cycle checkpoint kinases Chk1 and Wee1 in platinum resistant cell line. Overall miR-506-3p expression may recapitulate a BRCAness phenotype sensitizing EOC cells to chemotherapy and helping in selecting patients susceptible to DNA damaging drugs in combination with new small molecules targeting DNA-damage repair pathway.

## Introduction

In physiological conditions, DNA damage events constantly occur due to endogenous processes such as replication fork collapse or reactive oxygen species, as well as exogenous genotoxic agents such as UV/ionizing radiation or chemical agents capable to induce DNA lesions.

For the maintenance of genomic integrity and stability, cells have evolved a series of complex and coordinated mechanisms, overall defined as the DNA damage response (DDR) pathway (1). DDR comprises a network of proteins that constitutively sense DNA damage. Depending upon the specific type of damage detected, they signal to recruit other proteins for DNA repairing by a number of specific mechanisms (base-excision repair, mismatch repair, nucleotide-excision repair), which mostly involve Poly(ADP-Ribose) polymerase 1 (PARP1) catalytic activity. If a cell fails to repair single-strand DNA lesions, the consequent Double Strand Breaks (DSBs) lesions are preferentially repaired by Homologous Recombination repair (HR) and Non-Homologous End Joining (NHEJ) pathways. BRCA1/2, RAD genes (RAD51, RAD17), MRE11-RAD50-NBS1 (MRN) complex and Fanconi anemia genes (PALB2, FANCD2) are among the critical members of such pathways. Concurrently, upon DNA damage, ATR and ATM kinases are activated to coordinate cell cycle checkpoint signaling proteins such as Chk1 and Wee1 for halting the cell cycle progression to promote DNA repair, in order to minimize duplication of DNA-damaged cells (see for a review (1–3)).

Genomic instability is a well recognized cancer hallmark (4, 5) arising from defects in DDR pathways and oncogene-induced increased replicative stress, which are both common events in cancer. Such defects in DDR machinery, while increasing the genomic instability, originate a greater reliance of cancer cells on compensatory DDR pathways, which can be exploited as a therapeutic target for synthetically lethal approaches in anti-cancer therapy (6). Accordingly, inhibitors of PARP1 (PARPi) have been successfully used to induce the stalling of replication forks and consequently lethal DNA DSBs in a setting where another key component of the DDR pathway (BRCA1/2 gene) was already inactivated (7). PARPi have also proven their clinical efficacy, being approved for the treatment of BRCA1/2 mutated ovarian cancer patients but with clear evidence of benefit also in patients bearing homologous-recombination deficiency (HRD) (8).

Epithelial Ovarian Cancer (EOC) is a relatively low frequency disease. Nevertheless, it has one of the highest deaths to incidence ratio among the female tumor (59%) (9). Thus, improving survival and response to therapy is still a major challenge. EOC is characterized by late diagnosis, when the disease has spread throughout the abdominal cavity and the treatment paradigm is primary debulking surgery followed by platinum (Pt) based adjuvant therapy (10). In spite of a good response rate to front line therapy, the vast majority of EOC patients eventually relapse. In Pt-sensitive disease the use of PARPi as maintenance therapy clearly showed its efficacy by substantially improving progression free survival time (11). However, resistance to both Pt and PARPi frequently occurs (12) and represents the major issue that consistently limits the therapeutic opportunities for such patients (13). Exploitation of the synthetic lethal approach beyond the PARPi by both targeting DDR signaling dependent upon replicative stress and exploring combination therapies (12), is expected to offer new therapeutic approaches.

In cancer, the DDR signaling network is also affected by the altered expression of miRNAs that may ultimately impact on chemosensitivity by targeting DDR-related genes (14). We have identified and validated in different clinical settings a 35 miRNA-based predictor (MiROvaR) of early disease relapse in EOC (15–17), containing the ChrXq27.3 miRNA cluster, which we showed to be lost or downmodulated in early relapsing EOC patients (18) regardless the specific tumor histotype. We contributed to define miR-506-3p, the prominent member of the ChrXq27.3 miRNA cluster, as a key regulatory node of EOC cellular plasticity and response to chemotherapy (19, 20) and reported the association of miR-506-3p expression with sensitivity to Pt treatment in a cohort of 130 EOC patients (18, 20). By identifying RAD51 as a miR-506-3p target gene, we could directly link the effects of miR-506-3p expression on Pt-sensitivity to the DNA repair machinery and show its synthetic lethality with PARPi in EOC *in vitro* and pre-clinical *in vivo* models (20).

In the present study, we investigated the ability of miR-506-3p to target further members of the DDR cascade, besides RAD51, with the overall aim to identify additional mechanisms on which EOC cells rely for survival that, taking advantage of the synthetic lethality approaches, could be possibly exploited for specific therapeutic targeting.

## Material and Methods

### Cell Culture

Human EOC cell lines used in this study were: SKOV3 (TP53 null; obtained by ATCC), maintained in RPMI1640 (Lonza); OAW42 (TP53 wt; provided by Dr. A. Ullrich, Max-Planck Institute, DE), maintained in EMEM (Sigma-Aldrich) supplemented with 1% non-essential amino acids (Euroclone); CAOV3 (TP53mut; obtained by ATCC), maintained in DMEM (Lonza) supplemented with 25mM HEPES (Life Sciences); OV90 (TP53mut; obtained from ATCC) maintained in 1:1 MCDB-105 (Sigma-Aldrich) and Medium-199 (Sigma-Aldrich). The human cell line HEK293 (obtained by ATCC) was maintained in DMEM. All media were supplemented with 10% FBS (Lonza) and 2mM glutamine (Sigma-Aldrich). Cells were cultured at 37°C in a humidified atmosphere of 5% CO_2_ and subjected to short tandem repeat (STR) DNA profiling according to the manufacturer’s instructions and ATCC guidelines. Analyses were performed by our Genomic Facility at INT, Milan. Cells were routinely confirmed to be mycoplasma-free by a MycoAlertPLus detection kit (Lonza).

### Transient Transfection Assays

Cells were seeded into 6 well plates (Costar) at 200,000 cells/well and oligonucleotides transfection, including miRNA mimics, siRNAs and plasmids for luciferase assay, were performed using Lipofectamine 2000 (Thermo Fisher), according to the manufacturer’s instructions.

Ectopic expression of miR-506-3p was pursued by exposing EOC cells to 40nM of miRVANA miRNA mimic miR-506-3p and miRVANA miRNA scramble negative control (Thermo Fisher).

In RAD17 silencing experiments, cells were transfected with 40nM of siRNA molecules (siGENOME Smart Pool small interfering RNA, Dharmacon) or non-targeting siRNA (Dharmacon) as negative control. miRNAs and siRNA transfection efficiency were evaluated 48/72 h post transfection by assessing miRNAs and genes levels by qRT-PCR.

### Drug Treatments

The day after miRNAs/siRNA transfection, cells were exposed to drug treatments at indicated doses/times. In clonogenic assays Cis-platinum (Pt) (TEVA Italia) was used at doses ranging from 0.1 to 1μM. As described (21), this assay required a lower Pt concentration than that required for the DNA damage assay that is performed at Pt doses corresponding to the IC50 of each cell line as previously defined by TiterGlo or SRB assays (22). For SKOV3 cells also the sub-citotoxic IC30 Pt dose corresponding to 1μM was used. Chk1 inhibitor LY2603618 (Selleckchem) and Wee1 inhibitor MK1775 (Biovision) were reconstituted in dimethyl sulfoxide (DMSO) and used at doses ranging from 50 to 500nM and from 50 to 200nM, respectively. For all assays, drugs were diluted in cell culture media.

### Clonogenic Assay

After transfection/silencing, 2000 cells/well were seeded into 6 well plates in triplicate. Cells were then exposed to drug treatment and the ability of single cell to grow into a colony was evaluated after 10-14 days. The colonies were fixed using ice-cold methanol for 10 min, stained with 0.5% crystal violet solution (Sigma-Aldrich), and washed with distilled water. Colonies that contained more than 50 cells were counted using optical microscope.

### Immunofluorescence Staining

Cells were seeded on sterilized glass coverslips in 24 well plates (Thermo Fisher). Following treatments, cells were fixed in PBS-2% paraformaldehyde for 20 min, permeabilized with PBS-0.1% Triton X-100 (Sigma) for 10 min and saturated with PBS-1% BSA for 30 min. Cells were incubated with the primary antibody to γH2AX (see **Table 1**) at the concentration indicated in the datasheet and stained with the appropriated secondary antibody (Alexa Fluor^®^ 546-red, 1:1000 dilution). After washing twice, slides were mounted with ProLong Gold antifade reagent with DAPI (Invitrogen) for nuclei staining. For γH2AX staining experiments, scr/miR-506-3p transfected cells adhered to coverslips were treated with 1µM (CAOV3, OAW42, OV90) or 3µM (SKOV3) Pt for 24h. Images were acquired with the Leica TCS SP8 X confocal laser scanning microscope (Leica Microsystems GmbH, Mannheim, Germany) in the format 512 x 512 pixels in a single plane using a HC PL APO CS2 63X/1.30 oil-immersion objective and analyzed using Leica LAS AF rel. 3.3 (Leica Microsystems GmbH) software. Images were processed using Adobe Photoshop software. For micronuclei detection, after adhesion on coverslip, SKOV3 transfected cells were treated with 3µM Pt for 48h and 150ng/ml Nocodazole (Sigma-Aldrich) was added 8h before fixing for synchronization. Immunofluorescence was evaluated with a Nikon TE2000-S microscope with a 40X PlanFluor objective (Nikon). Images were acquired with ACT-1 software (Nikon).

**Table 1:** List of the antibodies used.

Antibody	Clone	Supplier
VINCULIN	4650	Cell Signaling	
β-ACTIN	A2066	Sigma-Aldrich	
RAD17	A305-788A-M	Bethyl Laboratories	
RAD51	H-92: sc-8349	Santa Cruz	
CHK1	2G1D5 2360	Cell Signaling	
pCHK1(S296)	D309F 90178	Cell Signaling	
γ-H2AX	A300-081A-M	Bethyl Laboratories	
WEE1	D10D2 13084	Cell Signaling	
pWEE1(S645)	D47G5 4910	Cell Signaling	
CDK1/CDC2	POH1 9116	Cell Signaling	
pCDK1/CDC2(Y15)	10A11 4539	Cell Signaling	
CYCLIN B1	D5C10 12231	Cell Signaling	
anti-GOAT IgG-HRP	sc-2354	Santa Cruz	
anti-MOUSE IgG-HRP	NA931V	GE Healthcare	
anti-RABBIT IgG-HRP	NA934V	GE Healthcare	
anti-RABBIT Alexa Fluor® 546-red		Invitrogen/Molecular Probes	

### Western Blot Analysis

Cells were washed with ice-cold PBS and directly lysed with NuPAGE LDS sample buffer (Thermo Fisher) under reducing conditions. Protein extracts were separated by SDS-PAGE using pre-casted NuPAGE Novex gels and blotted using iBlot2TM Dry Blotting System (Invitrogen). Membranes were rehydrated with TBS-T buffer [20mM Tris, 150mM NaCl, pH 7.6, 0.1% Tween 20] and saturated with 5% skim milk powder (Merck Millipore) in TBS-T. Membranes were incubated overnight at 4°C with the appropriate primary antibody and then incubated for 1h at room temperature with secondary HRP-antibodies (respective dilutions were specified by datasheets). See **Table 1** for the full list of antibodies used. Proteins were visualized using ECL chemo luminescence system (Bio-Rad); signals were acquired with a Bio-Rad apparatus using ChemiDoc XRS (Bio-Rad) and analyzed using Quantity One software (Bio-Rad).

### RNA Extraction and Quantitative Real-Time PCR (qRT-PCR)

Total RNA was extracted with NucleoSpin miRNA kit (Macherey-Nagel) following the manufacturer’s instruction, starting from cell pellets resuspended with TRIzol Reagent (Thermo Fisher). For total RNA reverse transcription, 2μg of total RNA were reverse transcribed to cDNA with High-Capacity cDNA Reverse Transcription Kit (Thermo Fisher). For miRNA reverse transcription, 10ng of total RNA was reverse transcribed to cDNA with TaqMan MicroRNA Reverse Transcription Kit (Thermo Fisher) using specific stem-loop reverse transcription primers and following the manufacturer’s instruction. All cDNA were stored at -20°C. qRT-PCR was performed using the 7900HT 00 system (Thermo Fisher) and TaqMan Fast Universal PCR Master Mix (Thermo Fisher), according to the manufacturer’s instructions. Specific RAD17 and hsa-miR-506-3p TaqMan FAM probes (Thermo Fisher) were used for gene amplification. GAPDH or RPL13A were used as stably expressed housekeepers for gene expression while RNU44 or RNU48 for miRNA expression. The ΔCT method was used to determine the quantity of the target sequences. See **Table 2** for the full list of qRT-PCR probes.

**Table 2:** List of the qRT-PCR probes used.

Probe	Code	Supplier
TaqMan™ Gene Expression Assay GAPDH	Hs03929097	ThermoFisher
TaqMan™ Gene Expression Assay RPL13A	Hs01926559	ThermoFisher
TaqMan™ Gene Expression Assay RAD17	Hs00267910	ThermoFisher
TaqMan™ Gene Expression Assay RAD51	Hs00153418	ThermoFisher
TaqMan™ MicroRNA Assay hsa-miR-506-3p	001050	ThermoFisher
TaqMan™ MicroRNA control Assay RNU44	001094	ThermoFisher
TaqMan™ MicroRNA control Assay RNU48	001006	ThermoFisher

### Luciferase Assay

A 25bp region of 3’UTR RAD17 gene containing the miR-506-3p seed region was cloned in the pmiR-Glo Dual Luciferase miRNA Target Expression Vector (Promega) according to the manufacturer instruction. The following sequences derived from the 3’UTR GAGTGTAAACTGTGTCCTTA (sense; in bold the seed region) and TAAGGCACACAGTTTACACTC (antisense) were used to generate the two oligos:

(5’-AAATAGCGGCCGCTACGAGTGTAAACTGTGTGCCTTATTTACT-3’ and

5’-CTAGAGTAAATAAGGCACACAGTTTACACTCGTAGCGGCCGCTATTT-3’), which included the restriction sites for cloning the fragment of 43-47bp into the vector. All clones were verified by DNA sequencing. For the luciferase assay, 5x10^4^ HEK293T cells were seeded in triplicate in 24 well plates and transfected with 1μg pmiRGlo vector together with 50nM miR-506-3p mimics, unrelated miR or scrambled miR as negative controls. Twenty-four hours after transfection, cells were lysed and luciferase activities were determined as for a dual-luciferase assay reporter system (Promega), according to the manufacturer’s instructions.

### Target Protector Experiments

miR-506-3p miRNA mimic or scrambled control miRNA were co-transfected with a target protector (TP) oligonucleotide (QIAGEN miScript Target Protector) specific to the conserved seed region of miR-506-3p within the 3′UTR of the RAD17 gene. The RAD17-TP was designed using Qiagen’s miRNA target protector design tool (www.qiagen.com/miDesign) using the RefSeq ID of RAD17 transcript variant 1 (NM_133338) as a reference template. Concentration of 40 and 60nM of TP were co-transfected with 60nM of miR-506-3p mimic or scrambled control miRNA. The efficiency of miRNA inhibition by the TP was measured by qRT-PCR on mRNA and by western blotting on lysates from transfected cells *vs*. controls.

### Cell Cycle Evaluation

Cells were collected and washed in PBS, fixed in ice-cold 70% ethanol and incubated with 10µg/ml RNAse A (Sigma-Aldrich) for 30 min. DNA was stained with Propidium Iodide (PI) 20µg/ml (Sigma-Aldrich) for 10 min before analysis. Stained cells were analyzed using a BD LSRII Fortessa instrument (BD Biosciences) and results analyzed using FlowJo software (Tree Star Inc).

### Statistical Analysis

Statistical analyses were carried out using GraphPad Prism software (version 5.02), as detailed throughout the manuscript. Asterisks in all figures denote a statistically significant difference in comparison with the relative control *P < 0.05; **P < 0.01; ***P < 0.001. Data reported are the mean ± S.D. of at least three independent experiments unless otherwise specified.

## Results

### MiR-506-3p Expression Controls Platinum Sensitivity and Sensing of DNA Damage

**Figure 1:**
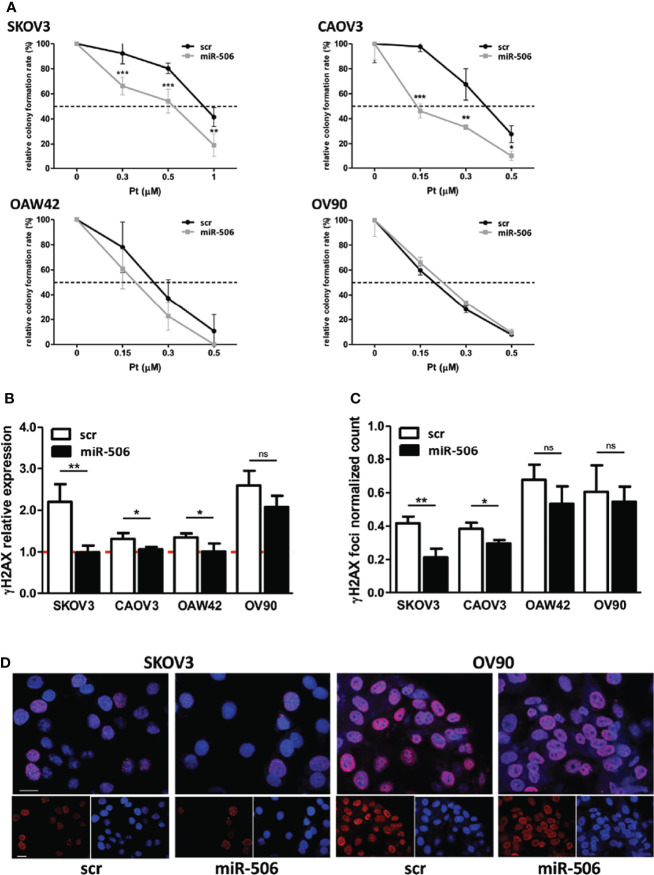
Forced expression of miR-506-3p increased platinum sensitivity in EOC cell lines and impaired sensing of DNA damage. **(A)** Clonogenic assay on EOC cell lines transfected with scrambled (scr) control miR or miR-506-3p (miR-506) mimic and treated with platinum (Pt) at the indicated concentrations. Curves were generated from two to four independent experiments, each one performed in triplicates. Two way ANOVA and Bonferroni’s post test was used for statistical analysis (*p<0.05; **p<0.01; ***p<0.001). **(B)** Assessment of relative γH2AX expression in EOC cells transfected with miR-506-3p mimic (miR-506) or scrambled (scr) control miR and treated with 1μM (CAOV3,OAW42, OV90) or 3μM (SKOV3) Pt corresponding to the IC50 of each cell line as defined by TiterGlo/SRB assay. For Western Blot analysis, lysates were collected at 24 hours after Pt-treatment; vinculin was used as loading control. Bars represent the ratio of γH2AX expression (normalized on vinculin) between Pt-treated and untreated cells (dashed red line represents γH2AX expression in untreated cells). Student’s t-test was used to compare miR-506 versus scr treated cells (* p<0.05; ** p<0.01; ns = not significant). Quantification **(C)** and representative images **(D)** of γH2AX foci in miR-506-3p/scr transfected OC cell lines. **(C)** Cells were treated as indicated in B and IF-stained with γH2AX and DAPI (nuclei). The percentage of γH2Ax foci/DAPI positive of treated *vs* untreated cells is reported. Student’s t-test was used to compare miR-506-3p versus scr treated cells (*p<0.05; **p<0.01; ns = not significant). **(D)** Representative confocal IF images of fixed SKOV3 and OV90 cells treated as described in panel B and stained with anti-γH2AX (red); nuclei were stained with DAPI (blue). Bars, 25 μm.

In a panel of EOC cell lines heterogeneous in terms of Pt sensitivity, histotype and TP53 status (23–25), we verified by clonogenic assays the impact of miR-506-3p expression on sensitivity to Pt treatment. Following Pt exposure, we observed that the colony formation ability of miR-506-3p transfected (miR-506) cells was significantly reduced as compared to their relative control (scr) in SKOV3 and CAOV3 cell lines, while the effect was lower or negligible in the other two models tested (OAW42 and OV90) which already showed Pt sensitivity in basal condition (**Figure 1A**).

Interestingly, following 24h Pt exposure we observed in all the models tested that the H2AX Ser-139 phosphorylation, referred to as γH2AX, increased in control (scr) cells as compared to their untreated counterpart, due to sensing of DNA damage. At variance, in miR-506-3p transfected-Pt-treated SKOV3, CAOV3 and OAW42 cells γH2AX resulted to be significantly reduced while in the OV90 cell line in spite of a comparable trend of reduction, the γH2AX level showed a greater variance (**Figure 1B**). Overall these data supported the notion that miR-506-3p expression could impair sensing of DNA damage. By counting γH2AX foci following Pt-treatment, we observed a significant inhibitory effect caused by miR-506-3p expression in the Pt-resistant cell lines (SKOV3 and CAOV3), while the Pt-sensitive cell lines (OAW42 and OV90) were affected by a greater variance (**Figure 1C**, for representative γH2AX foci images **Figure 1D**).

### RAD17 Is a Direct Target of MiR-506-3p and Associates With Worse Prognosis in EOC Patients

**Figure 2:**
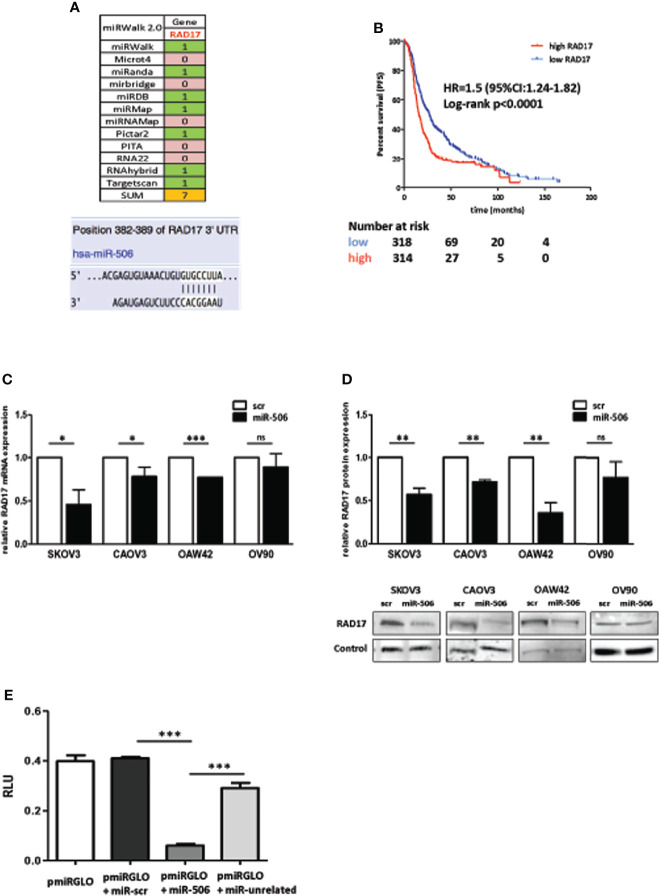
RAD17 is a target of miR-506-3p and associates with worse prognosis in EOC patients. **(A)** List of algorithm challenged with MirWalk2.0, with the prediction of miR506-3p targeting on *RAD17* 3’UTR. 0 = absence 1 = presence of a seed region for miR-506-3p in the 3’UTR of *RAD17*. Lower panel: images from microRNA.org and TargetScan prediction tools showing the alignment of miR-506-3p within the predicted binding site in the *RAD17* 3’UTR. **(B)** Kaplan-Meier survival curves of EOC patients stratified according to *RAD17* transcript expression. Data were extracted and analyzed with KM-plotter software (26). Progression free survival time was the clinical end-point analyzed. Data derived from 10 different publicly available datasets (GSE14764, GSE15622, GSE26193, GSE26712, GSE30161, GSE32062, GSE51373, GSE63885, GSE9891, TCGA) and selecting for patients treated with a Pt-based therapy. A total of 1291 patients were included in the analysis and stratified according to *RAD17* (207405_s_at probe) quartile expression. **(C, D).** Four EOC cell lines (SKOV3, CAOV3, OAW42, and OV90) were transfected with miR-506-3p mimic (miR-506) or scrambled control miR (scr). RAD17 expression was analyzed by qRT-PCR **(C)** to check *RAD17* mRNA and by Western blot **(D)** to check RAD17 protein expression levels. Bar charts represent the ratio of down-regulation of *RAD17* mRNA **(C)** or protein **(D)** expression in miR-506-3p versus scr transfectants taken as reference of expression for each cell line. RNU48 and RNU44 were used for normalization in qRT-PCR assay. For western blot analysis loading control was β-actin for SKOV3, OAW42, OV90 cells and vinculin for CAOV3 cells. Student’s t-test was used to compare miR-506 versus scr cells (*p<0.5; **p<0.01;*** p<0.001; ns = not significant). **(E)** Dual-Luciferase Reporter assay confirming RAD17 as a direct target of miR-506-3p. HEK293T cells were transfected with pmiRGLO empty vector (pmiRGLO, white bar), or pmiRGLO containing the putative binding site of miR-506-3p in the *RAD17* 3’UTR in combination with a scrambled miRNA (scr) (pmiRGLO+miR-scr, black bar), miR-506-3p (pmiRGLO+miR-506-3p, gray bar) and an unrelated miRNA (pmiRGLO+miR-unrelated, dotted bar). Results are the ratio (Firefly/Renilla) of Relative Luminometer Units (RLU). Student’s t-test was used to compare t-miR506 versus t-scr or t-miR-unrelated cells (*** p<0.001).

Given the known regulatory role of miRNAs on gene expression, we hypothesized that miR-506-3p could increase response to chemotherapy by targeting multiple key gene(s) involved in early DNA damage sensing. To shed light on this mechanism and to identify targets of miR-506-3p possibly involved in this process, we took advantage of the miRWalk 2.0 prediction algorithm that simultaneously analyze and retrieve information from 12 different prediction algorithms. According to the data obtained, seven of these algorithms identified a predicted binding site for miR-506-3p in the 3’UTR region of *RAD17* gene (**Figure 2A**).

RAD17 is an early sensor of DNA damage and according to this role, its expression and activity are expected to be involved in response to DNA damaging drugs. In this perspective, by exploring 10 publicly available EOC gene expression data sets from heterogeneous EOC case materials, we selected those patients who received Pt-based therapy (n=1291) and stratified them according to *RAD17* expression. We observed that patients with high *RAD17* expression had a significantly worse prognosis (HR= 1.5; 95%CI: 1.24-1.82; log-rank p <0.0001), with a shorter median progression free survival (PFS) time (15.8 months) as compared with those having low RAD17 expression and a median PFS time of 27 months (**Figure 2B**).

The regulatory effects of miR-506-3p on RAD17 expression was then evaluated in a panel of 4 different EOC cell lines in which we verified that transfection of miR-506-3p mimic induced down-regulation of both RAD17 transcript (**Figure 2C**) and protein (**Figure 2D**) expression. The luciferase reporter assay performed in HEK293T cells transfected with a report vector containing the RAD17 3’UTR with miR-506-3p seed region, showed that ectopic expression of miR-506-3p caused a decrease in luciferase activity as compared to controls (**Figure 2E**), thus demonstrating the direct targeting of RAD17 by miR-506-3p.

### MiR-506-3p-mediated RAD17 Downregulation is Involved in Chemosensitization of Pt-Resistant SKOV3 Cell Line

**Figure 3:**
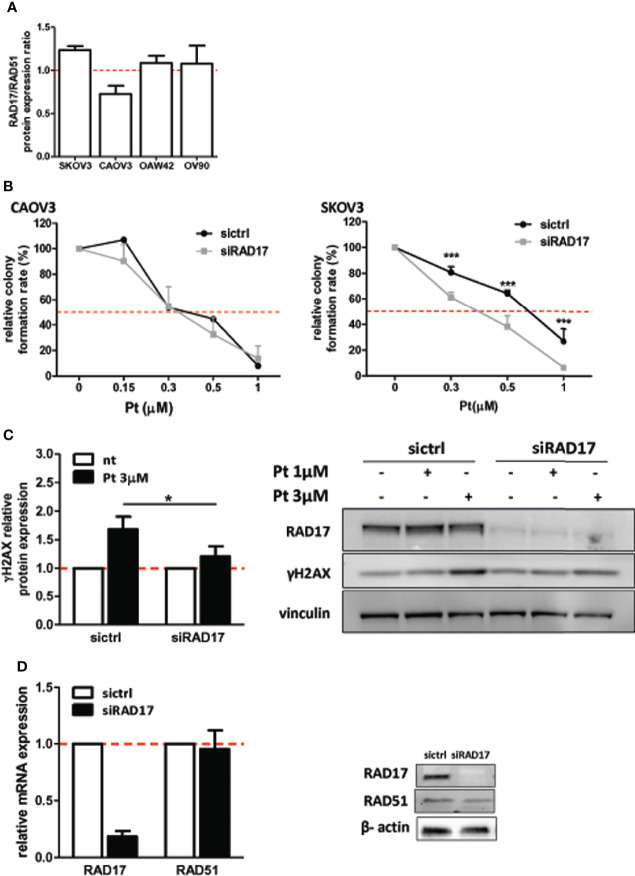
RAD17 silencing increased sensitivity to Pt-treatment in EOC in-vitro model. **(A)** Relative expression of RAD17 and RAD51 proteins as defined by Western Blotting on the panel of cellular models used. Bars represent the ratio of RAD17/RAD51. **(B)** Clonogenic assays of CAOV3 (left panel) and SKOV3 (right panel) cell lines silenced with siRNA targeting *RAD17* (siRAD17) or with a control siRNA (siCTRL) and treated with Pt at the indicated doses. Percentages of relative colony formation rate of siRAD17 versus siCTRL transfected cells are reported. Two way ANOVA and Bonferroni’s post test was used to compare groups (*** p<0.001). **(C)** Western blot analysis of γH2AX expression in SKOV3 cells silenced for RAD17 and treated with 1 and 3 µM Pt. Vinculin was used as loading control. *Left panel*: Bars represent the ratio of γH2AX expression (normalized on vinculin) between Pt-treated and untreated cells. Data are mean ± SD of at least three experiments. Student’s t test was used to compare siRAD17 cells versus sictrl transfected cells (*p<0.05); *right panel*: representative western blot. **(D)** Effects of RAD17 silencing on RAD17 and RAD51 mRNA (left panel) and protein (right panel) expression levels.

**Figure 4:**
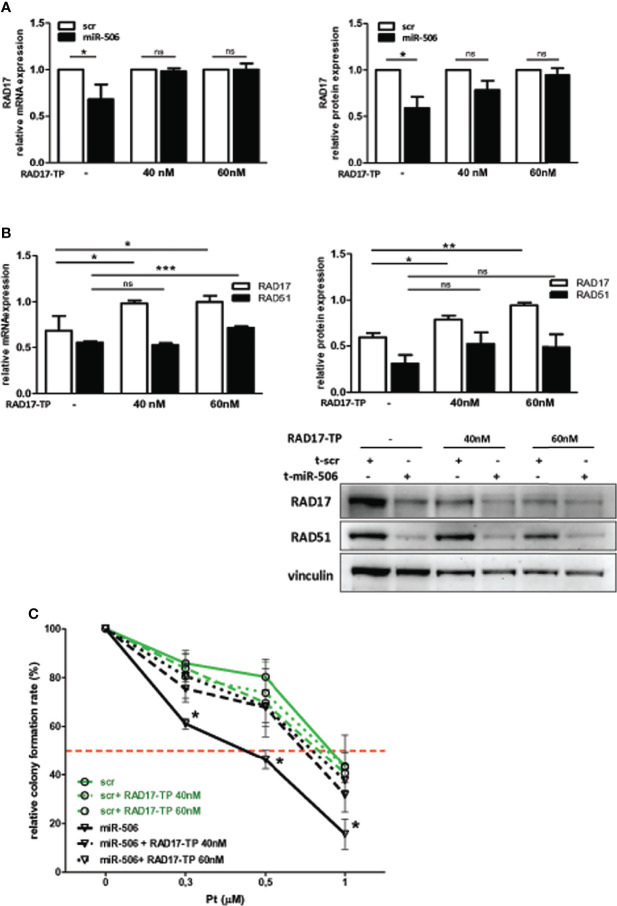
RAD17 regulation directly contributes to Pt-sensitivity mediated by miR-506-3p expression. **(A)** RAD17 quantification in t-miR506 and t-scr (control) SKOV3 cells co-transfected with RAD17 target protector (RAD17-TP). Two different concentration of RAD17-TP (40nM and 60nM) were tested. In the left panels is shown *RAD17* mRNA relative expression normalized on housekeeping genes and standardized on scr SKOV3 cells, while in the right panel is shown RAD17 protein quantification normalized to vinculin and standardized on scr SKOV3 cells (Student’s t test; *p<0.5; ns = not significant). **(B)** Efficacy of miR-506-3p targeting on RAD17 and RAD51 expression in the presence of RAD17-TP. Bars represent relative mRNA expression normalized on housekeeping gene (left panel) and relative protein expression quantified by western blot assays, normalized to vinculin and standardized on scr SKOV3 cells(upper panel). Student’s t-test was used (*p<0.5; **p<0.01; *** p<0.001; ns = not significant); lower right panel: representative western blot. **(C)** Percentage of relative colony formation rate following Pt treatment. SKOV3 cells were transfected with miR-506-3p (black lines) or scrambled miR (green lines) alone (solid lines) or each co-transfected with two doses of RAD17-TP (40nM dashed lines, 60 nM dotted lines) and then left untreated or treated with Pt at the indicated doses. Data are mean ± SD of two independent experiments, 6 replicates each. Two way ANOVA and Bonferroni’s post test was used to compare t-miR-506-3p versus t-miR-506-3p + 40/60nM RAD17-TP SKOV3 cells (* p<0.05).

miR-506-3p regulates the expression of 2 genes relevant for DNA damage repair, RAD51 [our previous observation (20)] and RAD17 (**Figure 2**) which could both contribute to Pt response possibly depending on their basal elative expression. All tested cell lines showed a balanced level of expression of RAD51 and RAD17 proteins with the exception of CAOV3 cells, expressing higher level of RAD51 (**Figure 3A**). To verify the role of RAD17 expression in response to Pt-treatment in the Pt-resistant SKOV3 and CAOV3 cell lines, we silenced *RAD17* and tested the response of siRAD17 cells to Pt exposure by clonogenic assay. We observed that, while CAOV3 cells were not affected by siRAD17 (**Figure 3B** left panel), supporting the notion of a greater contribution for RAD51, rather than RAD17, in the miR-506-3p-mediated chemosensitization for this cell line (see **Figure 1A**), the loss of RAD17 expression significantly increased SKOV3 sensitivity to drug treatment (**Figure 3B** right panel) phenocopying the effects obtained with miR-506-3p transfection (see **Figure 1A**). Furthermore, similarly to miR-506-3p transfection, in SKOV3 cells *RAD17* silencing caused a significant reduction of γH2AX as compared to the relative control cells following Pt treatment (**Figure 3C**). Finally, while transfection of miR-506-3p may affect expression of both RAD17 and RAD51, silencing of RAD17 did not affect RAD51 expression (**Figure 3D**). Given the multiple genes targeted by miR-506-3p and to further verify that the increase in Pt sensitivity caused by miR-506-3p in SKOV3 was also due to its direct regulatory effects on RAD17, we performed a target protector assay. We designed a single-strand modified RNA complementary to the miR-506-3p seed (RAD17-TP) in the 3’UTR of *RAD17* able to specifically compete with miR-506-3p for the binding. We co-transfected RAD17-TP with miR-506-3p in the SKOV3 cell line finding that, in the presence of RAD17-TP, miR-506-3p mimic lost its ability to down-regulate RAD17 expression both at mRNA (**Figure 4A**, left panels) and protein (**Figure 4A**, right panel) levels. To verify the specificity of RAD17-TP assay, we concurrently verified the effect on mRNA and protein expression of RAD51. Importantly, we observed that, in spite of a partial recovery of RAD51 mRNA at higher RAD17-TP dose (60nM), RAD51 protein expression was not substantially affected. Indeed, we observed that in presence of TP-RAD17, RAD51 protein expression continued to be inhibited in miR-506 transfected cells as compared to scr-transfected cells, overall supporting the selectivity of the system (**Figure 4B**). Notably, by exposing cells to Pt treatment, we observed by clonogenic assays that the co-transfection of RAD17-TP together with miR-506-3p was able to completely reverse in a dose-dependent manner the Pt sensitive phenotype caused by ectopic expression of miR-506-3p. Indeed, miR-506-3p/RAD17-TP co-transfected SKOV3 cells showed a two-fold increase in the IC50 (from 0.41 to 0.81 μM) as compared to SKOV3 transfected with miR-506-3p alone and displayed a dose-response curve comparable to those obtained in their relative controls (scr SKOV3 cells) which showed an IC50 comprised between 0.84 and 0.91 μM (**Figure 4C**). These results support the involvement of RAD17 among the multiple targets of miR-506-3p in controlling Pt sensitivity and confirm the specificity of the miR-506-3p/RAD17 regulatory axis in determining chemoresponse in EOC cell lines.

### MiR-506-3p Causes a Delay in G2/M Cell Cycle Arrest Induced by Pt Treatment and Impairs DNA Damage Signal Transduction Pathway

**Figure 5:**
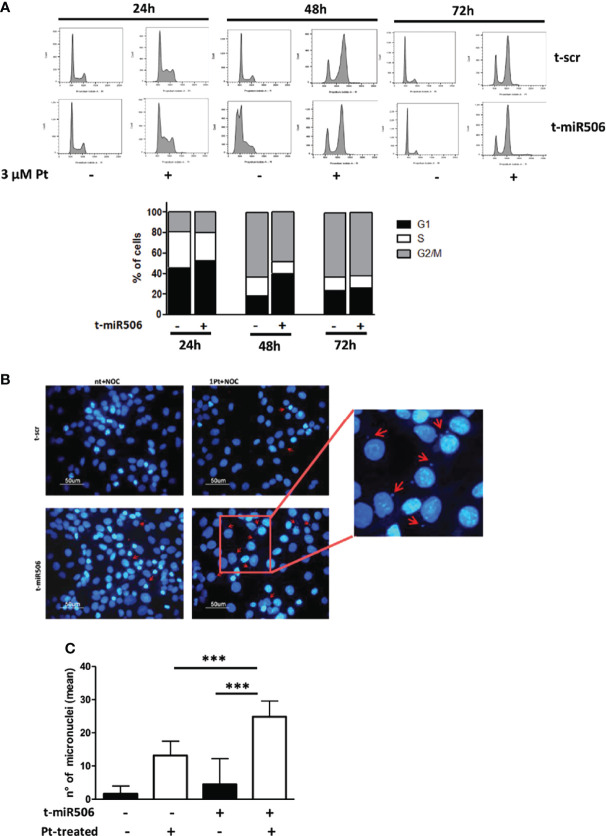
MiR-506-3p causes a delay in Pt-induced G2 cell cycle arrest and increases micronuclei formation. **(A)** Flow cytometry analysis of the cell cycle distribution (Propidium Iodide staining) in SKOV3 cells transfected with scrambled (scr) control miR or miR-506-3p mimic. Cells were left untreated or treated with 3μM Pt and cell cycle was analyzed at three time points (24, 48 72 hours). *Upper Panel:* Representative experiment; *Lower Pan*el: Percentage of Pt-treated cells in different cell cycle phases at the indicated time points as assessed by ModFit Analysis Software. Mean of three independent experiments is reported. Representative images **(B)** and quantification **(C)** of micronuclei structures in SKOV3 cancer cells labeled with DAPI. SKOV3 cells were transfected with miR-506-3p mimic or scrambled (scr) control miR and treated for 48h with 3µM Pt and nocodazole (150ng/ml). Micronuclei are indicated by red arrows. Data represent the mean ± SD of six different fields corresponding to two independent experiments (Student’s t test; *** p<0.001).

We then investigated the potential role of miR-506-3p in regulating progression of cell cycle in response to Pt treatment. We observed that following Pt exposure, miR-506-3p transfected cells showed a delay in accumulation in the G2/M phase of the cell cycle as compared to control scr-transfected cells. The effect was particularly evident at 48 hours upon Pt exposure, when in the G2/M phase we observed a 48% ( ± 10) of mir-506-3p transfected cells as compared to 63% ( ± 8) of scr-transfected control cells (**Figure 5A**). We therefore evaluated the presence of micronuclei, small extra-nuclear chromatin containing bodies resulting from unrepaired chromosome breaks or legging. Accordingly, following Pt treatment we observed by immunofluorescence analysis a significantly higher number of micronuclei in miR-506-3p transfected SKOV3 cells as compared to control scr transfected cells (**Figures 5B, C**).

**Figure 6:**
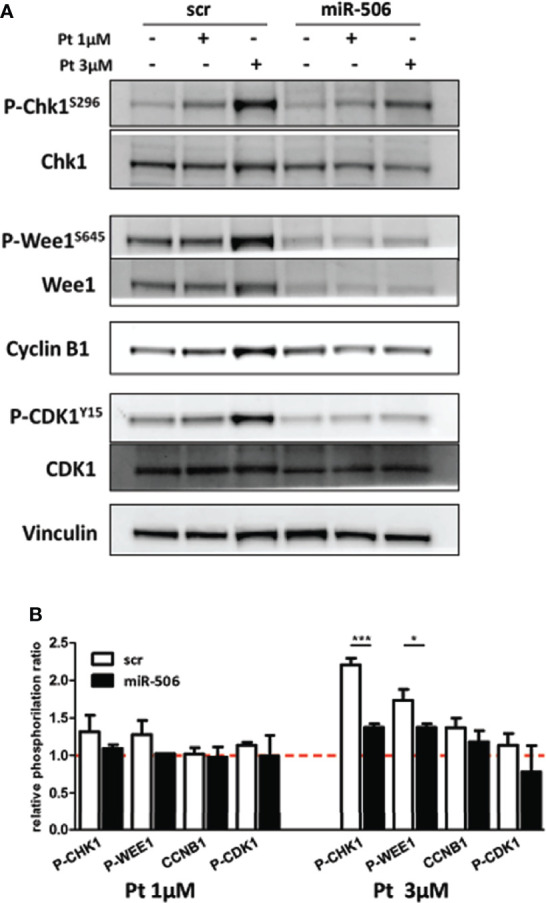
Abrogation of G2/M cell cycle checkpoint in miR-506-3p reconstituted cells. Western blot analysis on total cell lysates from SKOV3 cells transfected with miR-506-3p or scrambled (scr) miRs and following treatment with 1 or 3µM Pt. Immunoblottings were performed with antibodies against the proteins indicated. Vinculin was used as loading control. Representative western blot images **(A)** and quantification **(B)** of relative phosphorylation levels calculated as the ratio between phosphorylated and total protein normalized on loading control. Bars in the graph represent the phosphorylation ratio between Pt-treated and untreated cells. Only significant comparison assessed by Student’s t test are reported (* p<0.05; *** p<0.001).

These results prompted us to investigate if miR-506-3p reconstitution may cause major alterations in the signal transduction pathway activated upon DNA damage and related to cell cycle G2/M checkpoint activation. Following Pt treatment, in miR-506-3p reconstituted cells we observed a substantial decrease in both Chk1 and Wee1 phosphorylation as compared to control (scr) Pt-treated cells particularly at 3μM concentration (**Figure 6**). This inhibitory effect in turn reduced Cyclin-B1 activation and consequently CDK1 phosphorylation (**Figure 6**), possibly preventing the G2/M blockade in accordance with data obtained from cell-cycle analysis. Notably, we also observed a decrease of Wee1 protein (regardless of its phosphorylation status) in miR-506-3p transfected cells, which is suggestive of a possible indirect mechanism of regulation since Wee-1 is not listed among the predicted miR-506-3p target genes. Altogether, these data suggest that miR-506-3p expression, abrogating G2/M checkpoints in response to DNA damage, allows cells to enter in mitosis with an unrepaired extensive DNA damage.

### MiR-506-3p/RAD17 Axis Controls Sensing of DNA Damage and Potentiates Cell Cycle Checkpoint Inhibitors Activity

RAD17 has been recently shown to functionally interact with cell-cycle checkpoint kinases and its loss of function resulted to be synthetically lethal with Chk1 and Wee1 inhibitors in two cellular models with inactive p53 (27).

**Figure 7:**
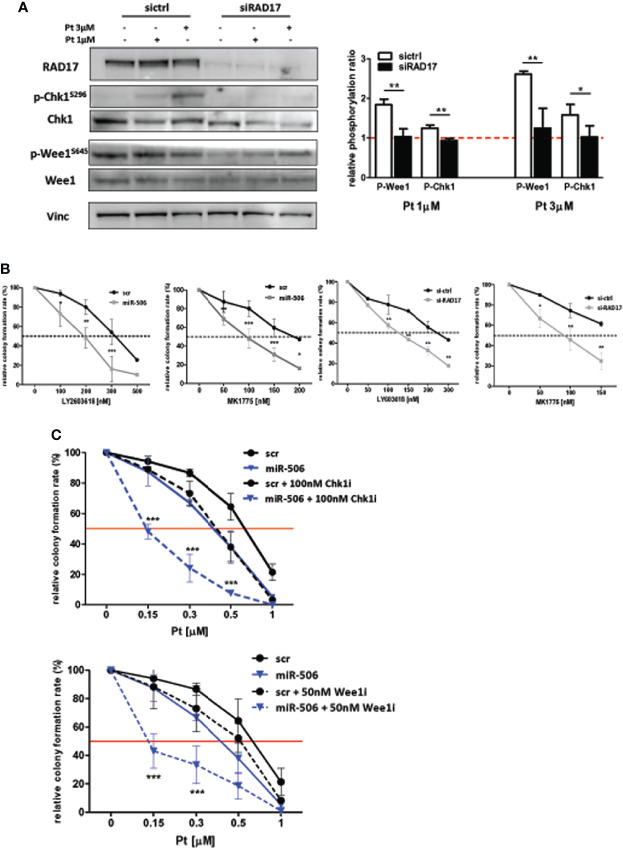
miR-506-3p dependent regulatory axis is synthetically lethal with cell-cycle checkpoint inhibitors. **(A)** Abrogation of G2/M cell cycle checkpoint activation in *RAD17* silenced SKOV3 cells. Western blot analysis on total cell lysates from SKOV3 cells silenced for *RAD17* (siRAD17) or with scrambled (sictrl) siRNA and treated or not with 1 and 3 µM Pt. Immunoblottings were performed with Abs against the proteins indicated. Vinculin was used as loading control. Representative western blot images (left panel) and quantification (right panel) of relative phosphorylation levels calculated as the ratio between phosphorylated and total protein normalized on loading control. Bars in the graph represent the phosphorylation ratio between Pt-treated and untreated cells. Comparison assessed by Student’s t test (* p<0.05; ** p<0.01). **(B)** Forced expression of miR-506-3p or *RAD17* silencing induces sensitivity to Chk1 and Wee1 inhibitors in SKOV3 cells. SKOV3 cells were transfected with miR-506-3p mimic or control scrambled miR (miR-506 and scr, left panels), or silenced with siRNA against *RAD17* or with a control siRNA (siRAD17 and sictrl, right panels) and tested by clonogenic assays for sensitivity to Chk1 inhibitor (LY603618) or Wee1 inhibitors (MK1775) at the indicated doses. **(C)** Wee1 or Chk1 inhibition in miR-506-3p reconstituted cells enhanced effect of Pt treatment. Both scr (black lines) and miR-506 (blue lines) SKOV3 cells were treated with Pt at the indicated doses, alone (solid line) or in combination with 100 nM Chk1 inhibitor (Chk1i; LY2603618, dotted lines, upper panel) or 50 nM Wee1 inhibitor (Wee1i; MK1775, dotted lines, lower panel). For each panel, percentages of relative colony formation rate are reported. To compare miR-506 or siRAD17 cells versus their relative control cells (*panel B*) or to compare miR-506 cells treated with Pt alone versus combinations with cell cycle checkpoint inhibitors (panel C), two way ANOVA and Bonferroni’s post test was used (*p<0.05; ** p<0.01;***p<0.001).

In accordance with the role of RAD17 in the DDR pathway, we observed also in our EOC *in-vitro* model that *RAD17* silencing, similar to miR-506-3p reintroduction (see **Figure 6**), caused a substantial decrease in Chk1 and Wee1 phosphorylation following exposure to 1 or 3 μM Pt (**Figure 7A**). Additionally, RAD17 silencing alone did not substantially affect OC cell growth as we observed only a 10.13± 5.6% of growth inhibition in three different experiments.

In line with the hypothesis of a direct miR-506-3p/RAD17 regulatory axis affecting DDR pathway, we sought to determine whether miR-506-3p expression, due to RAD17 targeting, could result lethal if combined with cell-cycle checkpoint inhibitors targeting Chk1 (LY2603618) or Wee1 (MK1775). By clonogenic assays we verified that the treatment with cell cycle checkpoint inhibitors alone (i.e. in t-scr or si-ctrl cells) resulted to have little effect on cell survival, which resulted to be affected only at high toxic doses (**Figure 7B**, black lines). Accordingly, we observed a remarkable sensitization to both Chk1 and Wee1 inhibitors in SKOV3 transfected with miR-506-3p mimic (**Figure 7B**, left panels) or silenced for RAD17 (**Figure 7B**, right panels) with a significant drop in the IC50 of both LY2603618 and MK1775 as compared to control cells. These observations further confirmed the convergence of miR-506-3p regulatory effects on mechanisms dependent also upon RAD17 expression.

Then we asked whether combination treatment strategies based on Pt and checkpoint kinase inhibitors could sensitize Pt-resistant cells to treatment. To this purpose we reconstituted miR-506-3p expression and treated SKOV3 cells with Pt alone or in combination with Chk1 and Wee1 inhibitors at a dose corresponding to their IC50. The data indicated that the combination of Pt with either LY2603618 100nM or MK1775 50nM, was more effective than Pt alone in reducing colony formation rate (**Figure 7C**). Furthermore, both the combined treatments showed a substantially enhanced effect in miR-506-3p transfected cells, causing a 3-fold reduction in the IC50 as compared to control (scr) cells (**Figure 7C**).

## Discussion

In this study, we demonstrated for the first time that the expression of miR-506-3p, the most well-known and studied miRNA belonging to the ChrXq27.3 miRNA cluster (18, 28), can sensitize EOC cells not only to DNA damaging drug but also to agent targeting cell cycle checkpoint proteins. miR-506-3p expression, through the direct regulation of RAD17 expression and function, reduces the ability of EOC cell to sense DNA damage and abrogates the G2/M cell cycle checkpoint. The consequent delay in the G2/M cell cycle arrest in response to DNA damage would eventually allow the entry into mitosis of heavily DNA-damaged cells, overall causing a sensitization to Pt treatment. Additionally, owing also to the action of miR-506-3p on RAD17, we have been able to identify a lethal combination of miR-506-3p expression with cell cycle checkpoint inhibitors.

Due to their regulatory role and pleiotropic effect, miRNAs affect most of the cellular processes. In cancer, their deregulated expression promotes the acquisition of cancer hallmark traits, not only leading to tumor development and progression, but also contributing to drug resistance (29–31). Most of the miRNAs included into the ChrXq27.3 miRNA cluster, whose retention we have shown to be associated to better EOC patients’ prognosis (18), have a tumor-suppressive role. Indeed, they are downregulated in various cancers and their expression is a clinically favorable prognostic factor (32). Specifically, miR-506-3p has various tumor-suppressive functions that we contributed to discover (33) associated with the epithelial–mesenchymal transition and proliferation. We also contributed to define the involvement of miR-506-3p in regulating RAD51 expression (20). However, a possible role for miRNA in influencing sensitivity to cell cycle checkpoint inhibitors has been so far poorly explored in EOC.

Our analyses in EOC cellular models of different origin showed that, particularly in Pt-resistant cell lines, ectopic expression of miR-506-3p was associated to a reduced ability to sense DNA damage detected as a reduced expression of γH2AX, the phosphorylated form of H2AX (34). Given the multi-target action of miRNAs which enable regulation of entire signaling networks, we explored the possibility of miR-506-3p to regulate genes other than RAD51 and involved in DNA repair and indeed, we have been able to validate also RAD17 as a miR-506-3p target. RAD17 is an early sensor of DNA damage that acts as a clamp loader for Claspin and the 9-1-1 complex, it is involved in the MRN complex recruitment (35–37) and it has been shown to regulate γH2AX formation (36). Acting both in the ATR-dependent signaling, related to maintenance of genomic stability, as well as in the ATM-related cascade (37), RAD17 contributes to controlling activation of DNA repair and DNA damage-associated replicative stress. According to this role, its expression and activity are expected to be relevant in response to DNA damaging drugs as well as cell cycle checkpoint inhibitors and its inhibition, possibly also through miRNA regulation, could therefore impair ability of cell to survive to DNA damage. Actually, we observed a survival advantage of Pt-treated EOC patients with decreased expression of RAD17 and *in vitro* the inhibition of RAD17 phenocopied the effects of miR-506-3p expression, increasing Pt sensitivity particularly in SKOV3 cell line, one of the most used EOC cellular model known for bearing inactivated p53 (TP53 null) and for being resistant to Pt and targeted treatments. The same Pt sensitization following RAD17 silencing was not observed in the other Pt resistant cell line CAOV3. Considering that miR-506-3p regulates both RAD51 and RAD17, the contribution of these molecules to Pt response following miRNA transfection, may be dependent on their relative expression and we have evidence of a grater RAD51 expression in CAOV3 as compared to SKOV3. The direct involvement of the miR-506-3p/RAD17 regulatory axis in determining response to Pt-treatment in SKOV3 was confirmed by the rescue of the resistant phenotype in the presence of a target protector that prevented RAD17 targeting by miR-506-3p.

Defective activation of cell cycle checkpoint and altered DNA repair capability cause high levels of replicative stress and accumulation of DNA damage, overall rendering cancer cells more sensitive to those compounds that exacerbate DNA damage process (3). This opens a possible therapeutic window beyond the use of PARPi, and expands the therapeutic landscape to those anti-tumor agents able to inhibit key mediators of DNA repair and replication, including Chk1 and Wee1 (38, 39). In this scenario, we highlighted the pivotal role in DNA damage signaling of RAD17 protein and recent literature demonstrated that RAD17 is a conserved key node for synthetic lethal interactions relevant for cancer therapy and specifically with cell cycle checkpoint kinases (40). Here we verified that, possibly through the inhibition of RAD17 expression, miR-506-3p expression could interfere with Chk1 and Wee1 inhibitors enhancing their activity. Although Wee1 is not a miR-506-3p predicted target, we observed its downregulation following miR-506-3p transfection. Noteworthy, among the described and validated miR-506-3p targets, there are CDK4 and CDK6 proteins (41), which regulate ATR expression in response to Pt treatment by stabilizing the transcription factor FOXO3 (42). Following this line, we can hypothesize an indirect effect mediated by transcription factor or other proteins targeted by miR-506 and acting upstream of Wee1, that will be worth to be investigated in an independent study.

These observations further support the relevance of the miR-506-3p-dependent regulatory axes and suggest new possible prognostic and therapeutic perspectives. From a clinical point of view, HRD has been correlated to better response to Pt derivatives and PARPi (8, 43, 44). Apart for BRCA1/2 mutations, it is now ascertained that genomic scars as well as genetic alteration or deficiencies in other HR-related genes are predictive of HRD and could positively affect response to Pt and PARPi treatment (45). By regulating RAD51 and RAD17, miR-506-3p significantly decreases the ability of tumor cells to repair drug-induced DNA damage overall increasing sensitivity to Pt, PARPi and cell cycle checkpoint kinases' inhibitors [(20) and present work.] Such effect strongly suggests a correlation of miR-506-3p expression with EOC *BRCAness* phenotype particularly for patients with a proficient BRCA status, and support further studies to verify its possible role in predicting response to therapy. Importantly, miR-506-3p expression also creates conditions for novel synthetic lethality approaches that could be possibly therapeutically exploited.

## Data Availability Statement

The original contributions presented in the study are included in the article/supplementary materials. Further inquiries can be directed to the corresponding authors.

## Author Contributions

MB and DM conceived and designed the study. RN performed most of the experiments on RAD17 as part of her PhD thesis. MV, AR, AN, and RM did the *in vitro* experiments. MB, DM, and AT critically revised all the data. All authors have been involved in interpretation of the results, reviewed the manuscript, and approved the final version. MB and DM provided funding.

## Funding

This study was supported by grants from the Italian Ministry of Health (5 x 1000 Funds—2013 to MB and RF 2016-02363995 to DM) and AIRC (IG17475 to DM).

## Conflict of Interest

The authors declare that the research was conducted in the absence of any commercial or financial relationships that could be construed as a potential conflict of interest.

## Publisher’s Note

All claims expressed in this article are solely those of the authors and do not necessarily represent those of their affiliated organizations, or those of the publisher, the editors and the reviewers. Any product that may be evaluated in this article, or claim that may be made by its manufacturer, is not guaranteed or endorsed by the publisher.

## References

[1] Jackson SP Bartek J. The DNA-Damage Response in Human Biology and Disease. Nature (2009) 461:1071–8. doi: 10.1038/nature08467 PMC2906700 19847258

[2] Roos WP Thomas AD Kaina B. DNA Damage and the Balance Between Survival and Death in Cancer Biology. Nat Rev Cancer (2016) 16:20–33. doi: 10.1038/nrc.2015.2 26678314

[3] Matthews HK Bertoli C de Bruin RAM. Cell Cycle Control in Cancer. Nat Rev Mol Cell Biol (2022) 23:74–88. doi: 10.1038/s41580-021-00404-3 34508254

[4] Hanahan D. Hallmarks of Cancer: New Dimensions. Cancer Discovery (2022) 12:31–46. doi: 10.1158/2159-8290.CD-21-1059 35022204

[5] Hanahan D Weinberg RA. Hallmarks of Cancer: The Next Generation. Cell (2011) 144:646–74. doi: 10.1016/j.cell.2011.02.013 21376230

[6] Pilié PG Tang C Mills GB Yap TA. State-Of-the-Art Strategies for Targeting the DNA Damage Response in Cancer. Nat Rev Clin Oncol (2019) 16:81–104. doi: 10.1038/s41571-018-0114-z 30356138 PMC8327299

[7] Farmer H McCabe N Lord CJ Tutt AN Johnson DA Richardson TB. Targeting the DNA Repair Defect in BRCA Mutant Cells as a Therapeutic Strategy. Nature (2005) 434:917–21. doi: 10.1038/nature03445 15829967

[8] Mirza MR Monk BJ Herrstedt J Oza AM Mahner S Redondo A. Niraparib Maintenance Therapy in Platinum-Sensitive, Recurrent Ovarian Cancer. N Engl J Med (2016) 375:2154–64. doi: 10.1056/NEJMoa1611310 27717299

[9] Sung H Ferlay J Siegel RL Laversanne M Soerjomataram I Jemal A. Global Cancer Statistics 2020: GLOBOCAN Estimates of Incidence and Mortality Worldwide for 36 Cancers in 185 Countries. CA Cancer J Clin (2021) 71:209–49. doi: 10.3322/caac.21660 33538338

[10] Lheureux S Gourley C Vergote I Oza AM. Epithelial Ovarian Cancer. Lancet (2019) 393:1240–53. doi: S0140-6736(18)32552-2 doi: 10.1016/S0140-6736(18)32552-2 30910306

[11] Longo DL. Personalized Medicine for Primary Treatment of Serous Ovarian Cancer. N Engl J Med (2019) 381:2471–4. doi: 10.1056/NEJMe1914488 31851805

[12] Gourley C Balmaña J Ledermann JA Serra V Dent R Loibl S. Moving From Poly (ADP-Ribose) Polymerase Inhibition to Targeting DNA Repair and DNA Damage Response in Cancer Therapy. J Clin Oncol (2019) 37:2257–69. doi: 10.1200/JCO.18.02050 31050911

[13] Ashworth A Lord CJ. Synthetic Lethal Therapies for Cancer: What’s Next After PARP Inhibitors? Nat Rev Clin Oncol (2018) 15:564–76. doi: 10.1038/s41571-018-0055-6 29955114

[14] Chowdhury D Choi YE Brault ME. Charity Begins at Home: non-Coding RNA Functions in DNA Repair. Nat Rev Mol Cell Biol (2013) 14:181–9. doi: 10.1038/nrm3523 PMC3904369 23385724

[15] Bagnoli M Canevari S Califano D Losito S Maio MD Raspagliesi F. Development and Validation of a microRNA-Based Signature (MiROvaR) to Predict Early Relapse or Progression of Epithelial Ovarian Cancer: A Cohort Study. Lancet Oncol (2016) 17:1137–46. doi: S1470-2045(16)30108-5 doi: 10.1016/S1470-2045(16)30108-5 27402147

[16] De Cecco L Bagnoli M Chiodini P Pignata S Mezzanzanica D. Prognostic Evidence of the miRNA-Based Ovarian Cancer Signature MiROvaR in Independent Datasets. Cancers (Basel) (2021) 13:1544. doi: 10.3390/cancers13071544 33801595 PMC8037414

[17] Ditto A De Cecco L Paolini B Alberti P Martinelli F Leone Roberti Maggiore U. Validation of MiROvaR, a microRNA-Based Predictor of Early Relapse in Early Stage Epithelial Ovarian Cancer as a New Strategy to Optimise Patients’ Prognostic Assessment. Eur J Cancer (2022) 161:55–63. doi: S0959-8049(21)01214-4 doi: 10.1016/j.ejca.2021.11.003 34922264

[18] Bagnoli M De Cecco L Granata A Nicoletti R Marchesi E Alberti P. Identification of a Chrxq27.3 microRNA Cluster Associated With Early Relapse in Advanced Stage Ovarian Cancer Patients. Oncotarget (2011) 2:1265–78. doi: 10.18632/oncotarget.3998 PMC3282083 22246208

[19] Sun Y Hu L Zheng H Bagnoli M Guo Y Rupaimoole R. MiR-506 Inhibits Multiple Targets in the Epithelial-to-Mesenchymal Transition Network and is Associated With Good Prognosis in Epithelial Ovarian Cancer. J Pathol (2015) 235:25–36. doi: 10.1002/path.4443 25230372 PMC4268369

[20] Liu G Yang D Rupaimoole R Pecot CV Sun Y Mangala LS. Augmentation of Response to Chemotherapy by microRNA-506 Through Regulation of RAD51 in Serous Ovarian Cancers. J Natl Cancer Inst (2015) 107:djv108. doi: 10.1093/jnci/djv108 25995442 PMC4554255

[21] Deng M Sun J Xie S Zhen H Wang Y Zhong A. Inhibition of MCM2 Enhances the Sensitivity of Ovarian Cancer Cell to Carboplatin. Mol Med Rep (2019) 20:2258–66. doi: 10.3892/mmr.2019.10477 PMC6691261 31322224

[22] Granata A Nicoletti R Tinaglia V De Cecco L Pisanu ME Ricci A. Choline Kinase-Alpha by Regulating Cell Aggressiveness and Drug Sensitivity is a Potential Druggable Target for Ovarian Cancer. Br J Cancer (2014) 110:330–40. doi: 10.1038/bjc.2013.729 PMC3899765 24281000

[23] Barnes BM Nelson L Tighe A Burghel GJ Lin IH Desai S. Distinct Transcriptional Programs Stratify Ovarian Cancer Cell Lines Into the Five Major Histological Subtypes. Genome Med (2021) 13:140,021-00952-5. doi: 10.1186/s13073-021-00952-5 34470661 PMC8408985

[24] Beaufort CM Helmijr JC Piskorz AM Hoogstraat M Ruigrok-Ritstier K Besselink N. Ovarian Cancer Cell Line Panel (OCCP): Clinical Importance of *In Vitro* Morphological Subtypes. PloS One (2014) 9:e103988. doi: 10.1371/journal.pone.0103988 25230021 PMC4167545

[25] Domcke S Sinha R Levine DA Sander C Schultz N. Evaluating Cell Lines as Tumour Models by Comparison of Genomic Profiles. Nat Commun (2013) 4:2126. doi: 10.1038/ncomms3126 23839242 PMC3715866

[26] Gyorffy B Lánczky A Szállási Z. Implementing an Online Tool for Genome-Wide Validation of Survival-Associated Biomarkers in Ovarian-Cancer Using Microarray Data From 1287 Patients. Endocr Relat Cancer (2012) 19:197–208. doi: 10.1530/ERC-11-0329 22277193

[27] Shen JP Srivas R Gross A Li J Jaehnig EJ Sun SM. Chemogenetic Profiling Identifies RAD17 as Synthetically Lethal With Checkpoint Kinase Inhibition. Oncotarget (2015) 6:35755–69. doi: 10.18632/oncotarget.5928 PMC4742139 26437225

[28] Yoshida K Yokoi A Sugiyama M Oda S Kitami K Tamauchi S. Expression of the Chrxq27.3 miRNA Cluster in Recurrent Ovarian Clear Cell Carcinoma and its Impact on Cisplatin Resistance. Oncogene (2021) 40:1255–68. doi: 10.1038/s41388-020-01595-3 PMC7892337 33420363

[29] Rupaimoole R Calin GA Lopez-Berestein G Sood AK. miRNA Deregulation in Cancer Cells and the Tumor Microenvironment. Cancer Discovery (2016) 6:235–46. doi: 10.1158/2159-8290.CD-15-0893 PMC4783232 26865249

[30] Si W Shen J Zheng H Fan W. The Role and Mechanisms of Action of microRNAs in Cancer Drug Resistance. Clin Epigenet (2019) 11:25,018-0587-8. doi: 10.1186/s13148-018-0587-8 PMC6371621 30744689

[31] Van Roosbroeck K Calin GA. Cancer Hallmarks and MicroRNAs: The Therapeutic Connection. Adv Cancer Res (2017) 135:119–49. doi: S0065-230X(17)30013-1 doi: 10.1016/bs.acr.2017.06.002 28882220

[32] Yoshida K Yokoi A Yamamoto Y Kajiyama H. ChrXq27.3 miRNA Cluster Functions in Cancer Development. J Exp Clin Cancer Res (2021) 40:112,021-01910-0. doi: 10.1186/s13046-021-01910-0 33766100 PMC7992321

[33] Sun Y Guo F Bagnoli M Xue FX Sun BC Shmulevich I. Key Nodes of a microRNA Network Associated With the Integrated Mesenchymal Subtype of High-Grade Serous Ovarian Cancer. Chin J Cancer (2015) 34:28–40. doi: 10.5732/cjc.014.10284 25556616 PMC4302087

[34] Polo SE Jackson SP. Dynamics of DNA Damage Response Proteins at DNA Breaks: A Focus on Protein Modifications. Genes Dev (2011) 25:409–33. doi: 10.1101/gad.2021311 PMC3049283 21363960

[35] Zou L Cortez D Elledge SJ. Regulation of ATR Substrate Selection by Rad17-Dependent Loading of Rad9 Complexes Onto Chromatin. Genes Dev (2002) 16:198–208. doi: 10.1101/gad.950302 11799063 PMC155323

[36] Wang Q Goldstein M Alexander P Wakeman TP Sun T Feng J. Rad17 Recruits the MRE11-RAD50-NBS1 Complex to Regulate the Cellular Response to DNA Double-Strand Breaks. EMBO J (2014) 33:862–77. doi: 10.1002/embj.201386064 PMC4194111 24534091

[37] Wang X Zou L Lu T Bao S Hurov KE Hittelman WN. Rad17 Phosphorylation is Required for Claspin Recruitment and Chk1 Activation in Response to Replication Stress. Mol Cell (2006) 23:331–41. doi: S1097-2765(06)00449-7 doi: 10.1016/j.molcel.2006.06.022 16885023

[38] Brown JS O’Carrigan B Jackson SP Yap TA. Targeting DNA Repair in Cancer: Beyond PARP Inhibitors. Cancer Discovery (2017) 7:20–37. doi: 10.1158/2159-8290.CD-16-0860 28003236 PMC5300099

[39] Lheureux S Mirza M Coleman R. The DNA Repair Pathway as a Target for Novel Drugs in Gynecologic Cancers. J Clin Oncol (2019) 37:2449–59. doi: 10.1200/JCO.19.00347 31403862

[40] Srivas R Shen JP Yang CC Sun SM Li J Gross AM. A Network of Conserved Synthetic Lethal Interactions for Exploration of Precision Cancer Therapy. Mol Cell (2016) 63:514–25. doi: S1097-2765(16)30280-5 doi: 10.1016/j.molcel.2016.06.022 PMC5209245 27453043

[41] Liu G Sun Y Ji P Li X Cogdell D Yang D. MiR-506 Suppresses Proliferation and Induces Senescence by Directly Targeting the CDK4/6-FOXM1 Axis in Ovarian Cancer. J Pathol (2014) 233:308–18. doi: 10.1002/path.4348 PMC4144705 24604117

[42] Dall’Acqua A Sonego M Pellizzari I Pellarin I Canzonieri V D’Andrea S. CDK6 Protects Epithelial Ovarian Cancer From Platinum-Induced Death *via* FOXO3 Regulation. EMBO Mol Med (2017) 9:1415–33. doi: 10.15252/emmm.201607012 PMC5623833 28778953

[43] González-Martín A Pothuri B Vergote I DePont Christensen R Graybill W Mirza MR. Niraparib in Patients With Newly Diagnosed Advanced Ovarian Cancer. N Engl J Med (2019) 381:2391–402. doi: 10.1056/NEJMoa1910962 31562799

[44] Coleman RL Fleming GF Brady MF Swisher EM Steffensen KD Friedlander M. Veliparib With First-Line Chemotherapy and as Maintenance Therapy in Ovarian Cancer. N Engl J Med (2019) 381:2403–15. doi: 10.1056/NEJMoa1909707 PMC6941439 31562800

[45] Miller RE Leary A Scott CL Serra V Lord CJ Bowtell D. ESMO Recommendations on Predictive Biomarker Testing for Homologous Recombination Deficiency and PARP Inhibitor Benefit in Ovarian Cancer. Ann Oncol (2020) 31:1606–22. doi: S0923-7534(20)42164-7 doi: 10.1016/j.annonc.2020.08.2102 33004253

